# 5α-Hydroxycostic acid inhibits choroidal neovascularization in rats through a dual signalling pathway mediated by VEGF and angiopoietin 2

**DOI:** 10.1186/s10020-023-00674-x

**Published:** 2023-11-01

**Authors:** Wulong Lei, Huan Xu, Hao Yao, Lanjiao Li, Menglei Wang, Xiyuan Zhou, Xueqin Liu

**Affiliations:** 1 Department of Ophthalmology, Second Affiliated Hospital of Chongqing Medical University, Chongqing, 400010 China; 2 Chongqing Key Laboratory of Ophthalmology, Chongqing, 400010 China

**Keywords:** Choroidal neovascularization, 5α-Hydroxycostic acid, VEGF, VEGFR2, Angiopoietin 2

## Abstract

**Background:**

5α-Hydroxycostic acid is a eudemane sesquiterpene that is isolated from the natural plant, *Laggera alata*. It exerts anti-inflammatory and anti-angiogenic effects on human breast cancer cells, but its role and underlying mechanism in choroidal neovascularization (CNV) are still unclear. We conducted a study to verify that 5α-Hydroxycostic acid can inhibit the formation and leakage of CNV, and describe the possible dual pathway by which it exerts its inhibitory effects in this process.

**Methods:**

An in vitro model of choroidal neovascularization was established using VEGF164, while a rat model of choroidal neovascularization was established using a 532 nm laser. In both models, the effects of 5α-Hydroxycostic acid in vivo and in vitro were evaluated to determine its inhibitory effect on abnormal cell proliferation, migration and tubule formation, as well as its effect on pathological changes in choroidal tissues and the area of neovascularization leakage in rats. The levels of components in the VEGF/VEGFR and Ang2/Tie2 signaling pathways were measured in tissues and cells.

**Results:**

In vitro experiments have shown that 5α-Hydroxycostic acid can inhibit abnormal cell proliferation, migration and angiogenesis. Additionally, 5α-Hydroxycostic acid enhances cell adhesion by inhibiting the phosphorylation pathways of VEGFR2 and Tie2. In vivo experiments demonstrated that 5α-Hydroxycostic acid has a positive therapeutic effect on choroidal neovascularization in rats. It can effectively reduce vascular leakage, consistent with the results of the cell experiments.

**Conclusion:**

5α-Hydroxycostic acid can inhibit choroidal neovascularization by interfering with the VEGF- and Ang2/Tie2-related pathways, and it may be a good candidate drug for treating CNV.

## Introduction

Age-related macular degeneration (AMD) is a leading cause of blindness among elderly individuals. Choroidal neovascularization (CNV) is the major cause of irreversible visual impairment in patients with AMD (Miller et al. 2021; Jonas et al. 2017; Fleckenstein et al. 2021; Ferrington et al. 2020), and its pathogenesis is complex (Wong et al. 2014; Seddon 2017). Although anti-vascular endothelial growth factor (VEGF) is the primary approach used to treat CNV, some patients do not respond to VEGF treatment (Rofagha et al. 2013; IVAN Study Investigators et al. 2012). Therefore, it is necessary to identify new drugs that can simultaneously target VEGF and other pathways.

VEGF plays a crucial role in the process of neovascularization. Targeted anti-VEGF therapy is a classic approach currently used to treat the formation of tumors (Hosaka et al. 2020). However, researchers are searching for new therapeutic targets due to the lack of response or adverse reactions in some patients. Angiopoietin plays an important role in blood vessel formation, remodeling, maturation and maintenance. The main functions of angiopoietin 1 include inhibiting endothelial cell apoptosis, promoting endothelial cell budding and stabilizing blood vessels (Witzenbichler et al. 1998). Meanwhile**,** angiopoietin 2 competitively inhibits Ang1, thus preventing the formation of unstable and leaky blood vessels. Increasing evidence indicates that Ang2 is closely related to tumor invasion, metastasis and angiogenesis, and promotes the occurrence and development of cancer via various mechanisms (Lind et al. 2005; Helfrich et al. 2009).

The Ang/Tie2 pathway is responsible for regulating vascular homeostasis, vascular permeability, neovascularization, and inflammation. Ang2 inhibits the phosphorylation of Tie 2 and antagonizes the effect of Ang1, leading to vascular instability and making blood vessels more susceptible to the effects of VEGF and other proinflammatory cytokines (Akwii et al. 2019). The VEGF and Ang/Tie2 pathways interact in antitumor responses and abnormal angiogenesis. Previous studies by Yvonne Kienast et al. showed that the anti-Ang2-VEGFA cross-reactive mAb, a novel bispecific human IgG1 antibody, inhibits the hematopoietic spread of tumor cells to other organs (Schmittnaegel et al. 2017; Felcht et al. 2012). Foxton et al. demonstrated in previous ophthalmic animal studies that anti-VEGFA/Ang2 combination therapy reduces choroidal neovascularization and subretinal inflammatory cell infiltration compared to anti-VEGFA monotherapy (Regula et al. 2019).

In our study, we found that a novel natural drug called 5α-Hydroxycostic acid can inhibit the abnormal migration and proliferation of rat choroidal endothelial cells. In a laser-induced rat model of CNV, 5α-Hydroxycostic acid effectively inhibited neovascularization and leakage. Both in vitro and in vivo studies have shown that 5α-Hydroxycostic acid can increase the expression of intercellular compact junction protein (ZO-1) and vascular endothelial cadherin (VE-cadherin) in the treatment of CNV. 5α-Hydroxycostic acid interferes with the VEGF and Ang2 pathways, strengthening intercellular junctions and reducing blood vessel leakage.

## Materials and methods

### Animals

A total of 80 male brown Norwegian rats aged 8 to 10 weeks were housed in the Animal Center of Chongqing Medical University and provided with standard food and water. All experiments were conducted in accordance with the Guide to the Care and Use of Experimental Animals and were approved by the Animal Research Ethics Committee of Chongqing Medical University (No. 134, 2022). The rats were randomly divided into four groups: the control + 0.2% dimethyl sulfoxide (DMSO) group, the CNV + 0.2% DMSO group, the CNV + 5α-Hydroxycostic acid group, and the CNV + Semaxinib group. All groups, except for the control, were subjected to the CNV model. Before establishing the model, all animal protocols were approved by the Institutional Animal Care and Use Committee.

### Culture and treatments of rat choroidal vascular endothelial cells (RCSECs)

RCSECs were purchased from BeNa Culture Collection and cultured in Dulbecco’s modified Eagle medium (DMEM) supplemented with 10% fetal bovine serum (FBS, HyClone Logan UT, USA), 100 U/mL penicillin, and 100 μg/mL streptomycin (all from Gibco, Thermo Fisher Scientific, Waltham, MA, USA). The vector group was treated with 0.2% dimethyl sulfoxide in medium, and the cells were maintained at 37 °C in a humidified incubator containing 5% CO_2_. Cells between the 3rd and the 10th generations were used in all experiments.

### Cytotoxicity test

The cytotoxicity of various concentrations of 5α-Hydroxycostic acid was assessed using the Cell Counting Kit-8 (Dojindo, Kumamoto, Japan). RCSECs were seeded in 96-well plates at a density of 1 × 10^4^ cells/well. After the cells attached to the wells, they were incubated with different concentrations of 5α-Hydroxycostic acid dissolved in DMSO for 48 h. Afterwards, Cell Counting Kit-8 (CCK8) reagent was added and incubated with the RCSECs for an additional 2 h. The absorbance was measured at a wavelength of 450 nm.

### Cell proliferation experiment

RCSECs were seeded in 96-well plates at a density of 1 × 10^4^ cells per well, and the inducible factor VEGF164 (30 μM) was added. After the cells attached to the wells, 5α-Hydroxycostic acid at concentrations of 25 μM, 50 μM, and 100 μM was added and incubated for 48 h. Following incubation, CCK8 reagent was added and incubated with the RCSECs for an additional 2 h, and absorbance was measured at a wavelength of 450 nm.

### Cellular scratch wound assay

RCSECs were cultured to 90% confluence in 6-well plates. A wound, 1 mm in width, was created in each well with a 200 μl pipette tip. After the detached cells were removed by washing with PBS, the cells were treated with medium containing 50 μM 5α-Hydroxycostic acid and VEGF164. Images were captured using an inverted microscope after incubation for 0 and 24 h.

### Transwell migration assay

RCSECs were adjusted to a concentration of 3 × 10^5^ cells/mL, and 600 µL of medium supplemented with different treatments was added to the subchambers of Transwell plates according to the different groups. Then, 100 µL of RCSECs in serum-free medium were added to the upper chamber and incubated in the incubator for 24 h. The culture medium from the upper chamber was discarded, and the non-migrated cells on the surface of the chamber were removed with a cotton swab. The migrated cells were then fixed with paraformaldehyde for 30 min, stained with crystal violet for 25 min, washed with PBS three times, and dried naturally. Three fields of the same membrane were randomly selected to capture photos (100×) under an inverted microscope. The relative vertical mobility was calculated as follows: relative vertical mobility = intervention group/control group.

### Tubule formation assay

RCSEC cells (1 × 104 cells/well) were seeded onto a Matrigel substrate (356234, BD Biosciences, Corning) and incubated for 6 h. Lumen formation was observed under an optical microscope, and the tubular structure was analyzed and quantified using ImageJ software.

### Real-time RT (reverse transcription)-PCR

Total mRNA was isolated from RCSECs (TRIzol Plus, Thermo Fisher, Waltham, MA, USA) using the chloroform method and eluted in 20 μl of diethyl pyrocarbonate (DEPC)-treated water. Complementary DNA was generated by reverse transcription polymerase chain reaction (RT-PCR) using Superscript III (Thermo Fisher, Waltham, MA, USA). Reverse transcription was performed at 58 °C for 50 min, and the enzyme was thermally inactivated at 85 °C. The real-time quantitative RT-PCR™ SYBR Green master mix was used with PowerTrack (Thermo Fisher, Waltham, MA, USA). The sequences of primers are shown in Table 1 and were synthesized by Tsingke Biotechnology Co. Ltd. (Beijing, China). Using β-actin as an internal reference, the relative quantitative values of target gene expression were determined, and statistical analysis was performed using the 2^−ΔΔCT^ method.

**Table 1:** Primer base sequence

Gene	Forward (5′–3′)	Reverse (5′–3′)
Ang-2	AGCACAAAGGATTCGGACAATGA	CCCTTCCAGTAGTACCACTTGAT
Tie2	GCTCTGGGAGATCGTTAGCTTAG	TCCCTCCAGATTGTCTCATTAG
VEGFR2	AGTGAAAGAGATGCGGGAAACTA	TCTTCTAGCTGCCAGTACCATTG
ZO-1	GAGAGGAAGAGCGCATGCTAAA	GCTGTCCGACTTGAGCATATACA
VE-Cadherin	ACAGAGGCCAATACTTCCGAATAA	CTGGGCAGCATTCTCACATACTT
β-Actin	AGATCAAGATCATTGCTCCTCCT	ACGCAGCTCAGTAACAGTCC

### Western blotting analysis

Total protein was extracted from RCSECs and rat choroid tissues. The samples were gently washed with PBS three times and then incubated for 15 min in lysis buffer. Total proteins were collected by centrifugation at 12,000×*g* for 10 min, and the precipitates were discarded. The proteins (20 µg) were separated by SDS-polyacrylamide gel electrophoresis and transferred to PVDF membranes. The nonspecific binding sites were blocked with TBST supplemented with 10% skim milk powder at 37 °C for 1 h, and then the membranes were incubated with specific antibodies at 4 °C overnight. Anti-β-actin antibodies (1:10,000, Affinity, China) were used as the internal control. After incubation with a secondary antibody, an enhanced chemiluminescent reagent (BioTech, Beijing, China) was used to detect the antibodies.

### Cellular immunofluorescence assay

The cells in each group were cultured on slides for 48 h, fixed in 4% paraformaldehyde (PFA) at room temperature for 20 min, blocked with 10% BSA at room temperature for 1 h, and incubated with primary anti-ZO1 antibodies (1:200; Proteintech, China) overnight at 4 °C. The cells were then washed with PBST and incubated at room temperature for 2 h with a FITC-conjugated fluorescent secondary antibody. After staining with DAPI for 5 min, an anti-fluorescence quenching agent was used to seal the nuclear stain. An inverted fluorescence microscope was used for image acquisition.

### Rat choroidal neovascularization (CNV) model

The rats in each group were anesthetized by intraperitoneal injection of Pentobarbital sodium (30 mg/kg, Xingguang Chemical Glass, Chongqing), after which a solution of compound tobramycin was used to dilate the pupils of the eyes. After flattening the eyes with a cover glass, the optic disc was located. A 532 nm krypton laser (power of 140 mW, diameter of 100 µm, and exposure time of 0.07 s) was used to perform 2PD centered on the optic disc, and 7 points were photocoagulated. Bubble generation was used as a sign that the Bruch film had been broken, and modeling was successful. On the 14th day after modeling, the rats in the CNV + 5α-Hydroxycostic acid group and CNV + Semaxinib group were anesthetized, and 5α-Hydroxycostic acid (50 µg/L, 10 µL/eye, Topscience, Shanghai Taoshu Technology Biology Co., Ltd.) and Semaxinib (4 µM, 10 µL/eye, Targetmol, America) were injected separately into the vitreous cavity. The rats in the other two groups were injected with the same volume of 0.2% DMSO. The rats were sacrificed by an overdose of anesthesia 14 days after injection, and then, the retina and choroid tissues were collected for subsequent experiments.

### Choroidal flat mount

Fluorescein angiography was performed 14 days after laser photocoagulation to confirm the successful establishment of the model. The rats were anesthetized with Pentobarbital sodium (30 mg/kg) on the 14th day after injection; the heart was exposed, and blood was drained from the right atrium using normal saline from the right ventricle. Additionally, the rats were injected with 4% paraformaldehyde for fixation and then injected with high-molecular-weight glucuronium conjugated to fluorescein isothiocyanate (MW: 100000, Xi'an Ruixi Biological Co., Ltd.) until the lips and limbs of the rats appeared fluorescent yellow. The eyeballs, anterior segment tissue, and vitreous body were removed, and the choroidal scleral complex was carefully separated for tiling. Fluorescence microscopy (Leica, Germany) was used to observe CNV lesions, and the CNV-related neovascularization area was measured using ImageJ image processing software.

### Haematoxylin eosin staining of tissue sections

The eyeballs of randomly selected rats from each group were harvested on the 14th day after intravitreal injection for paraffin sectioning. Paraffin sections with intact and continuous retinal and choroidal structures were selected and incubated in a 60 °C incubator for 20 min. A xylene solution was used for dewaxing, and 75%, 95%, and 100% alcohol solutions were used for dehydration. After washing, the sections were stained with hematoxylin for 5 min. After washing again, 10 g/L acid liquor was used for color separation, and ammonia was incubated for 15 s to return the color to blue. The sections were then washed with distilled water 3 times, stained with eosin for 8 min, and washed with distilled water 3 times. The degree of damage to the choroidal structure was observed under a microscope.

### Statistical analysis

All data are presented as mean ± standard deviation. Statistical significance between groups was assessed using a two-tailed unpaired Student t-test or ANOVA for comparison of two or multiple groups (GraphPad Prism, USA). Differences were considered significant when P < 0.05. All experiments were repeated at least three times.

## Results

### 50 µM 5α-Hydroxycostic acid can effectively inhibit VEGF164-induced cell proliferation

**Fig. 1:**
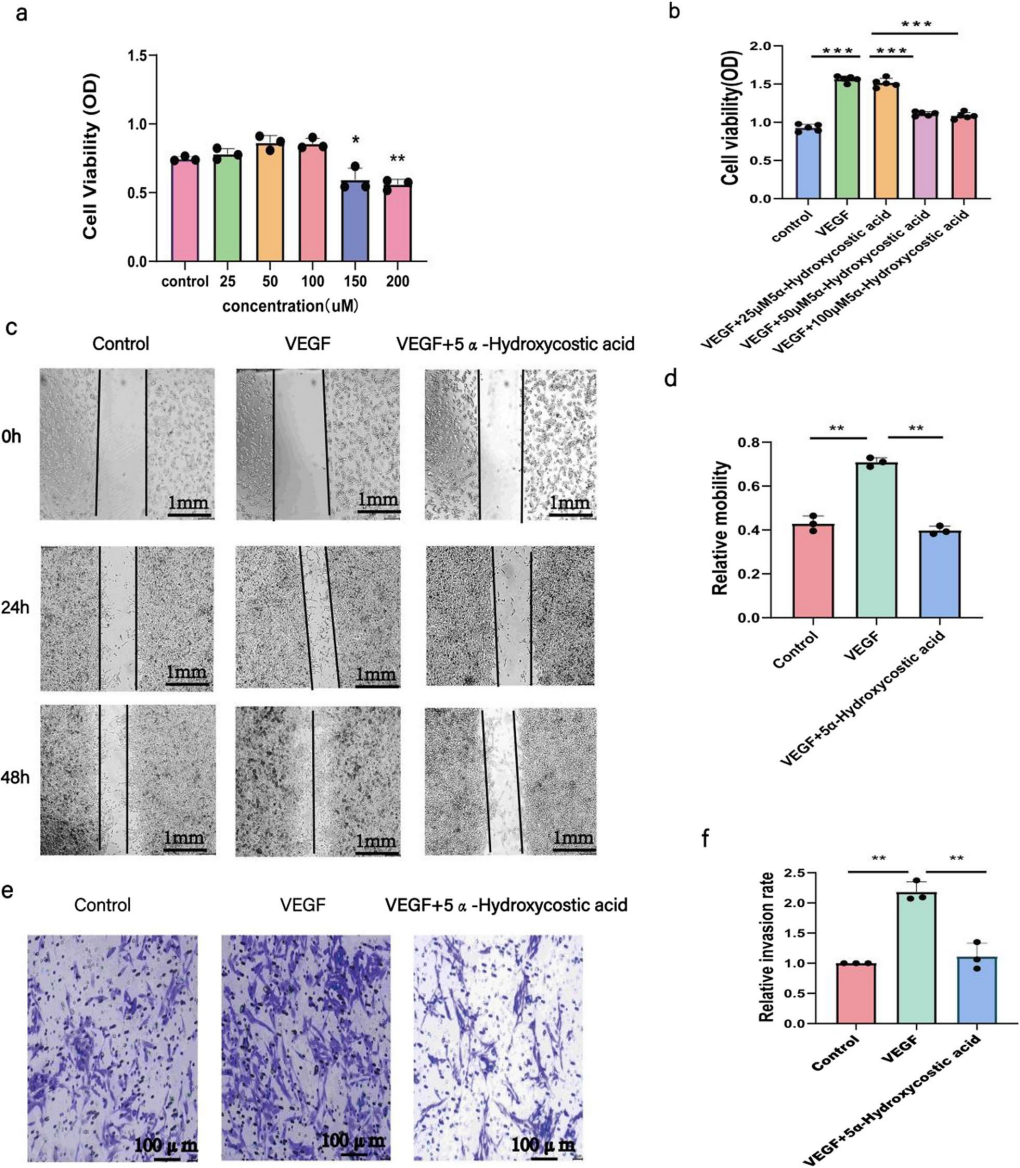
Effects of appropriate concentration of 5α-Hydroxycostic acid on VEGF-stimulated RCSECs migration. **a**, **b** Optimum concentration of 5α-Hydroxycostic acid action determined by Cell Counting Kit-8 method (50 µM). **c**–**f** The effects of 5α-Hydroxycostic acid on cell migration were detected by cell scratch assay and transwell assay (magnification, ×100). The results are presented as the mean ± SD. n = 3; *P < 0.05, **P < 0.01, ***P < 0.001

The CCK8 method was used to evaluate the toxic effects of 5α-Hydroxycostic acid on RCSECs (Fig. 1a). When the drug was dissolved in DMSO and the concentration was equal to or less than 100 µM, there was no toxic effect on the cells. However, at concentrations higher than 100 µM, the drug had an obvious effect on cell activity. When we stimulated RCSECs with a concentration of 30 ng/mL VEGF164, there was no significant difference between the cells treated with 50 µM and 100 µM 5α-Hydroxycostic acid and the control group, but cell viability was significantly reduced compared to the model group (Fig. 1b). There was no significant difference between the two concentrations of 5α-Hydroxycostic acid in terms of inhibiting VEGF-induced cell proliferation, so the optimal concentration of 5α-Hydroxycostic acid was determined to be 50 µM.

### 5α-Hydroxycostic acid effectively prevents abnormal cell migration

We aimed to investigate whether 5α-Hydroxycostic acid could effectively inhibit the abnormal migration of RCSECs without affecting the normal migration of cells. In the wound healing test, we clearly observed that 5α-Hydroxycostic acid had a significant inhibitory effect on VEGF164-induced RCSEC migration, as illustrated in Fig. 1c, d. Upon treatment with VEGF164, the cells migrated to the center of the wound, yet with the addition of 5α-Hydroxycostic acid, the cell migration was weaker than that of the stimulated group and resembled that of the control group. Furthermore, in the Transwell migration experiment, cell aggregates migrated through the 8.0 μm pore in the upper chamber in the VEGF164-treated group; however, after the addition of the drug, only scattered cells migrated to the lower chamber, and this result was similar to that of the untreated group, with no significant difference, but significantly different from that of the VEGF-164-treated group (Fig. 1e). These results indicate that 5α-Hydroxycostic acid has a good inhibitory effect on VEGF164-induced cell migration.

### 5α-Hydroxycostic acid exerts an inhibitory effect on the abnormal lumen formation of cells

**Fig. 2:**
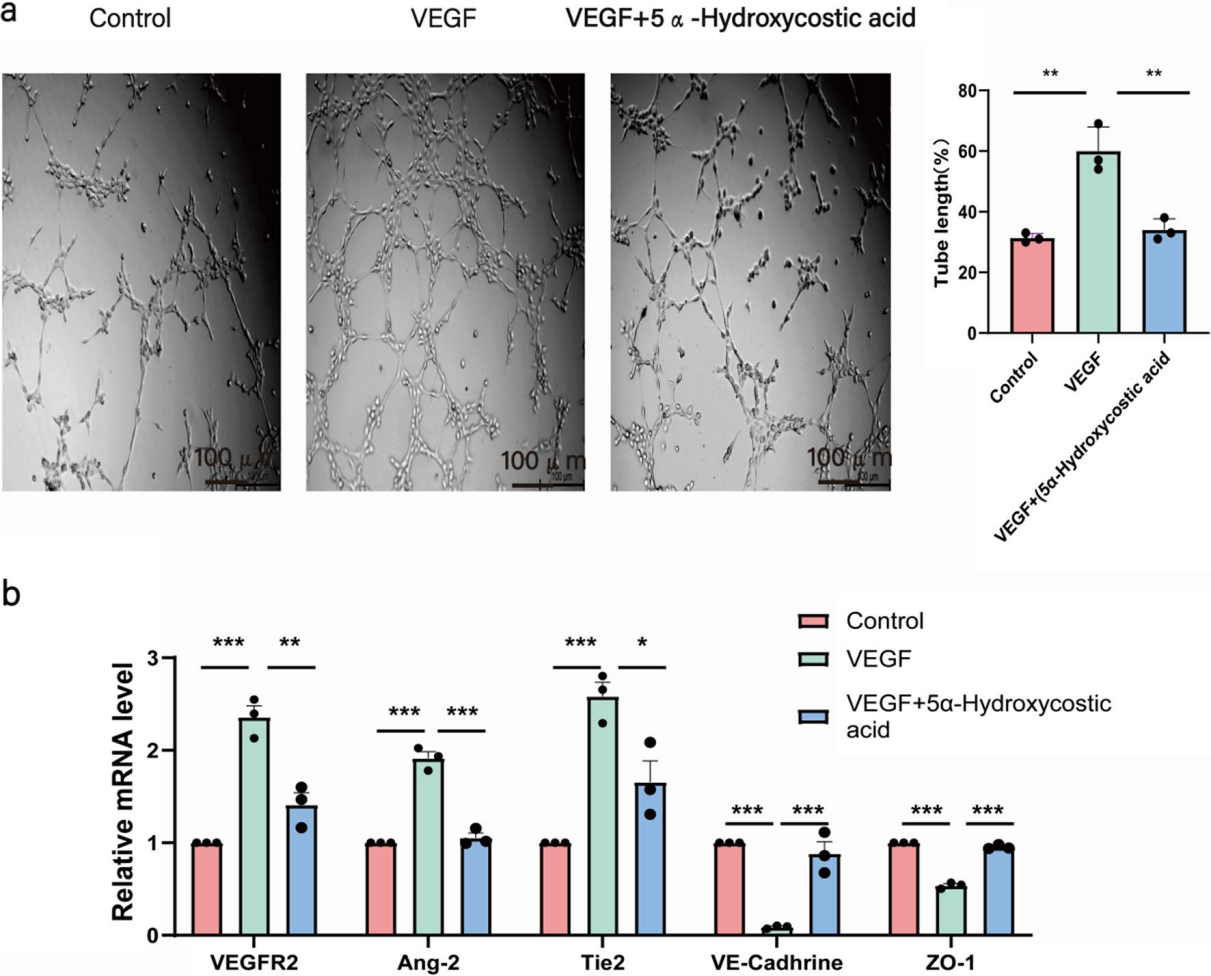
5α-Hydroxycostic acid inhibited VEGFR2, Ang2, Tie2 mRNA levels and increased VE-cadherin, ZO-1 mRNA levels. **a** Inhibition of VEGF-induced RCSECs tube formation by 5α-Hydroxycostic acid.Cell tubular structures were captured (magnification, ×100) and tube length was quantified (n = 3). **b** mRNA expression levels in control group, VEGF group, and VEGF + 5α-Hydroxycostic acid group. The results are presented as the mean ± SD. n = 3; *P < 0.05, **P < 0.01, ***P < 0.001

The formation of new microvessels through the migration of endothelial cells is a crucial step in choroidal neovascularization. Therefore, we investigated whether 5α-Hydroxycostic acid could inhibit VEGF-induced vascular lumen formation. Upon treatment with VEGF164, a relatively complete grid-like lumen formed between RCSECs. However, after treatment with 5α-Hydroxycostic acid, the tube formation was discontinuous. The tube formation in the 5α-Hydroxycostic acid-treated group differed significantly from that in the VEGF164-treated group and resembled that in the control group (Fig. 2a). The data indicate that 5α-Hydroxycostic acid effectively suppresses VEGF-induced tubulogenesis, suggesting its potential to inhibit angiogenesis.

### 5α-Hydroxycostic acid inhibits angiogenesis by the dual pathways of VEGF/vegfr and Ang2/Tie2

**Fig. 3:**
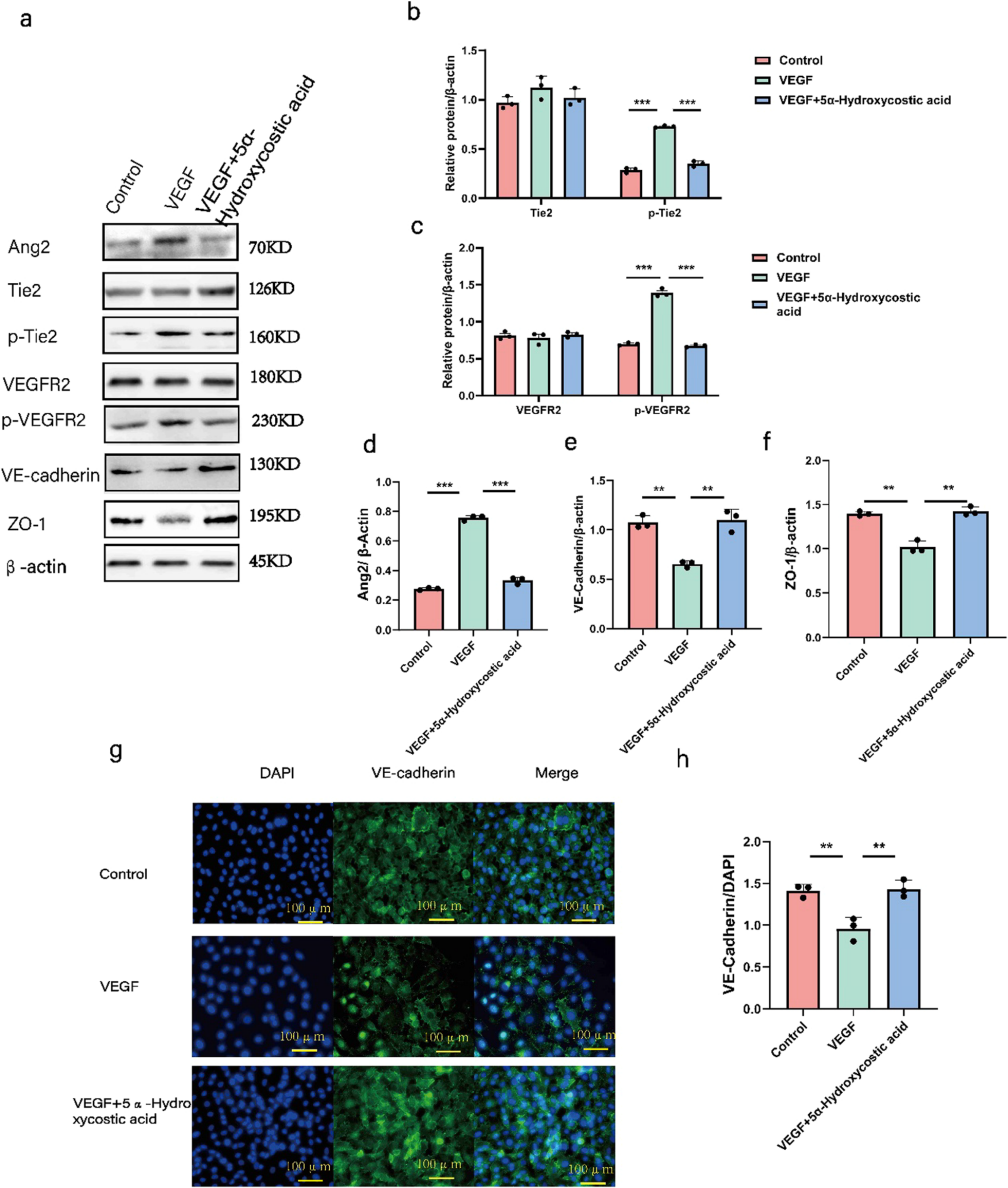
5α-Hydroxycostic acid inhibits angiogenesis by reducing the production of angiogenic factors to enhance intercellular adhesion. **a**–**f** The relative protein levels of VEGFR2, p-VEGFR2, Ang2, Tie2, p-Tie2, VE-cadherin and ZO-1 in each group were detected by Western blot. **g**, **h** Immunostaining analysis of VE-cadherin in three groups of cells (magnification, ×200). The results are presented as the mean ± SD. n = 3; *P < 0.05, **P < 0.01, ***P < 0.001

**Fig. 4:**
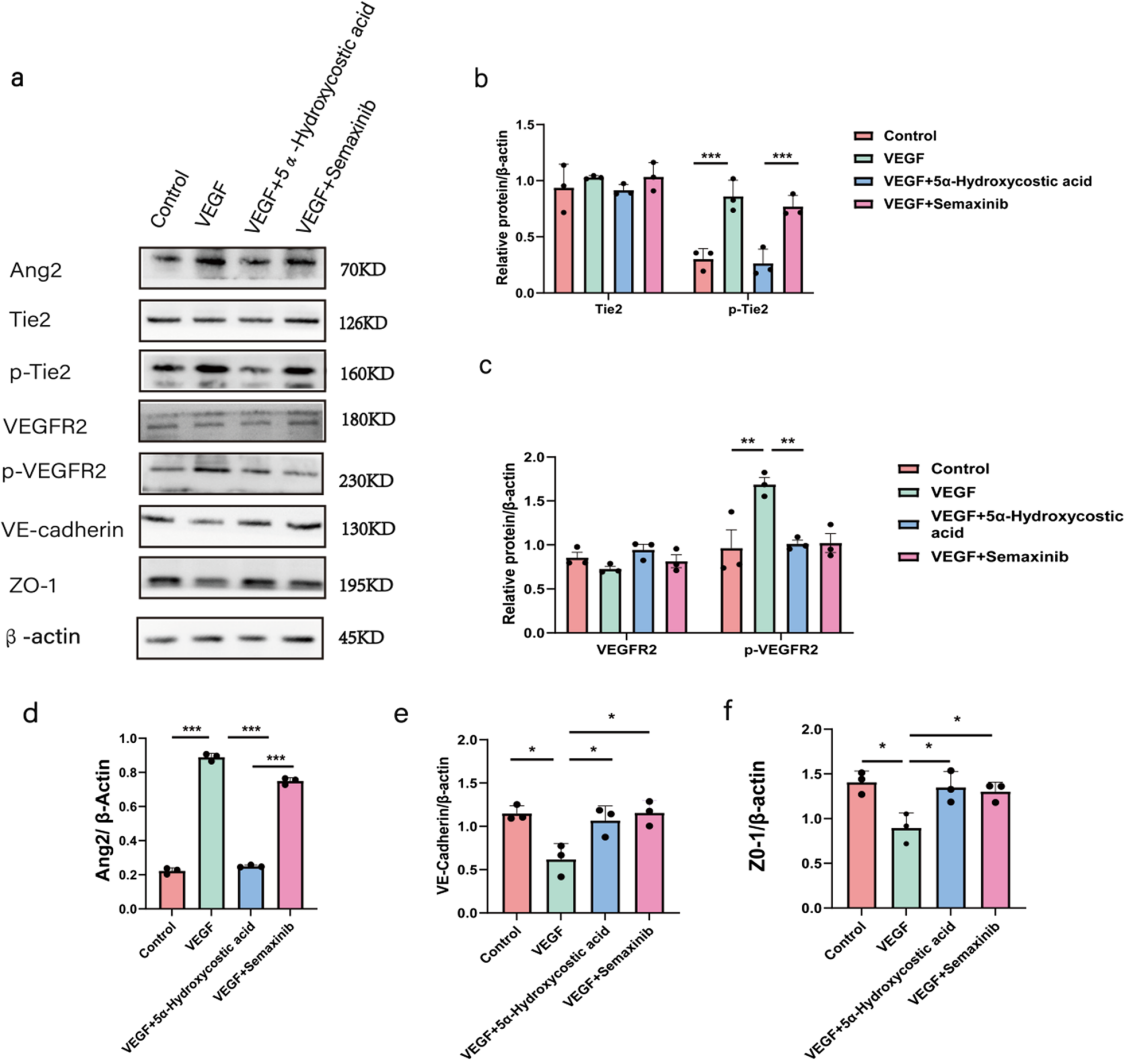
5α-Hydroxycostic acid inhibits angiogenesis by the dual pathways of VEGF/VEGFR and Ang2/Tie2. **a**–**c** The relative protein levels of VEGFR, Tie2, and their phosphorylated proteins in four groups were detected by Western blot. **d**–**f** The relative protein levels of Ang2, VE-cadherin and ZO-1 in each group were detected by Western blot. The results are presented as the mean ± SD. n = 3; *P < 0.05, **P < 0.01, ***P < 0.001

Next, we investigated the mechanism by which 5α-Hydroxycostic acid suppresses the VEGF164-induced migration and angiogenesis of RCSECs. Firstly, we evaluated the effect of 5α-Hydroxycostic acid on the expression of some angiogenesis-related genes in RCSECs by real-time PCR analysis. RCSECs were cultured in medium supplemented with 30 ng/mL VEGF164 and 50 µM 5α-Hydroxycostic acid for 48 h, and the mRNA expression of three vital angiogenic factors, namely, VEGFR2, Ang2, and Tie2, as well as cell adhesion-related genes, specifically VE-Cadherin and ZO-1, were determined. 5α-Hydroxycostic acid significantly reduced the mRNA level of VEGFR2, Ang2, and Tie2 induced by VEGF, while protein levels of cell adhesion-related genes were significantly increased (Fig. 2c). This finding suggests that 5α-Hydroxycostic acid inhibits VEGF-164-induced RCSEC angiogenesis by reducing the mRNA expression of VEGFR and Ang2/Tie2. Concurrently, we found that 5α-Hydroxycostic acid could significantly inhibit the phosphorylation of VEGFR2 and Tie2 in VEGF-164-stimulated cells, while there was no significant difference between the non-phosphorylated VEGFR2 and Tie2 groups (Fig. 3a–c). The expression of Ang2, VE-Cadherin, and ZO-1 was also significantly increased (Fig. 3d–h), enhancing cell adhesion, reducing vascular leakage, and playing a critical role in vascular stability. After adding a VEGF receptor protein tyrosine kinase inhibitor (Semaxinib), the levels of Ang2 and phosphorylated Tie2 were significantly higher in the group treated with Semaxinib than in the 5α-Hydroxycostic acid-treated group (Fig. 4). This result demonstrates that 5α-Hydroxycostic acid inhibits angiogenesis through dual pathways of VEGF/VEGFR and Ang2/Tie2.

### Choroidal neovascularization fundus lesions in rats

**Fig. 5:**
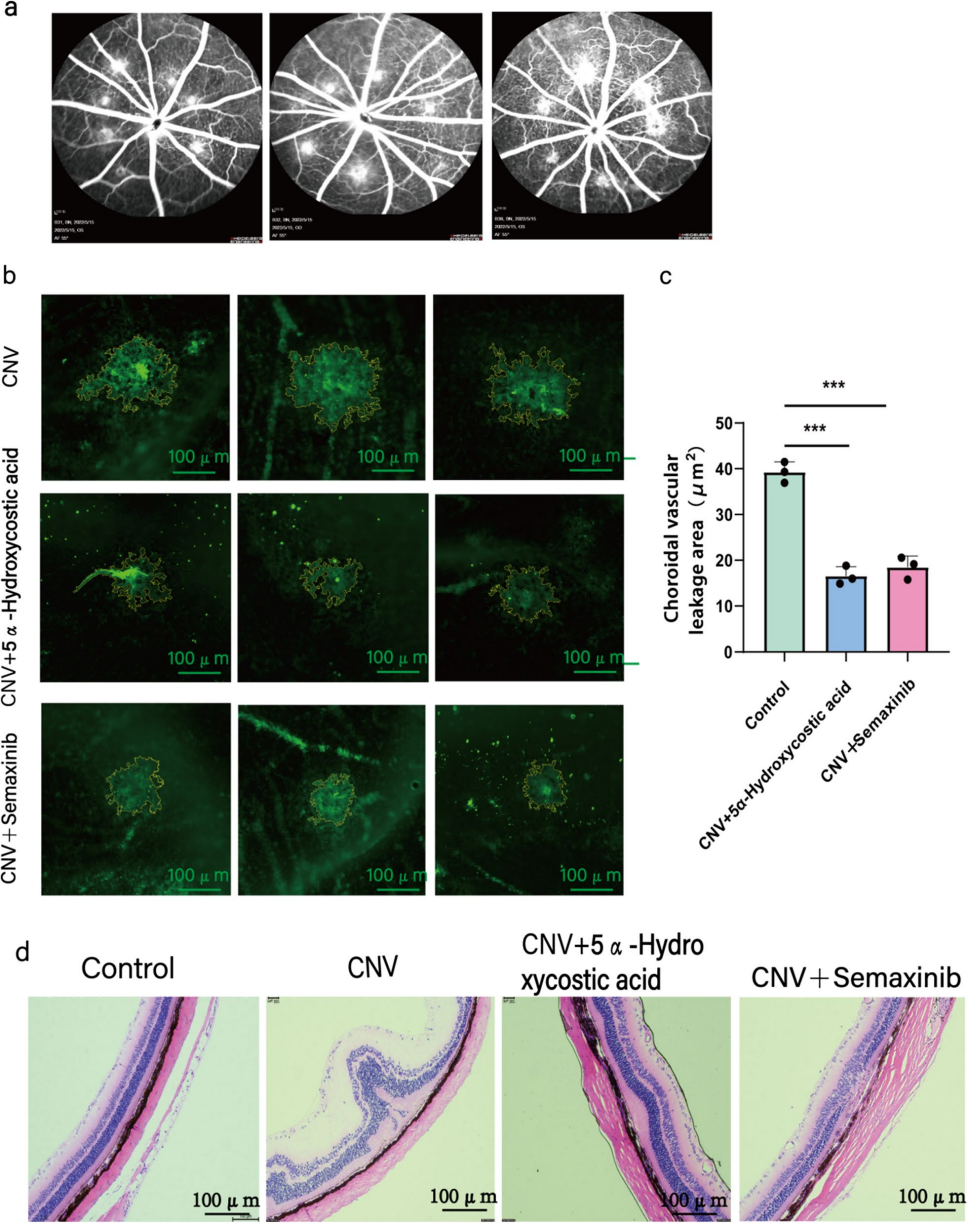
5α-Hydroxycostic acid can treat rat choroidal neovascularization and reduce neovascularization and leakage. **a** At day 14 after photocoagulation, fundus fluorescein angiography (FFA) was performed to evaluate CNV formation. **b**, **c** Choroidal patches and the area of choroidal neovascular leakage in CNV group, 5α-Hydroxycostic acid treatment groupand Semaxinib treatment group (magnification, ×200). **d** Hematoxylin–eosin (HE) staining observed the structural changes of retina and choroid in rats after treatment (magnification, ×100). The results are presented as the mean ± SD. n = 3; ***P < 0.001

**Fig. 6:**
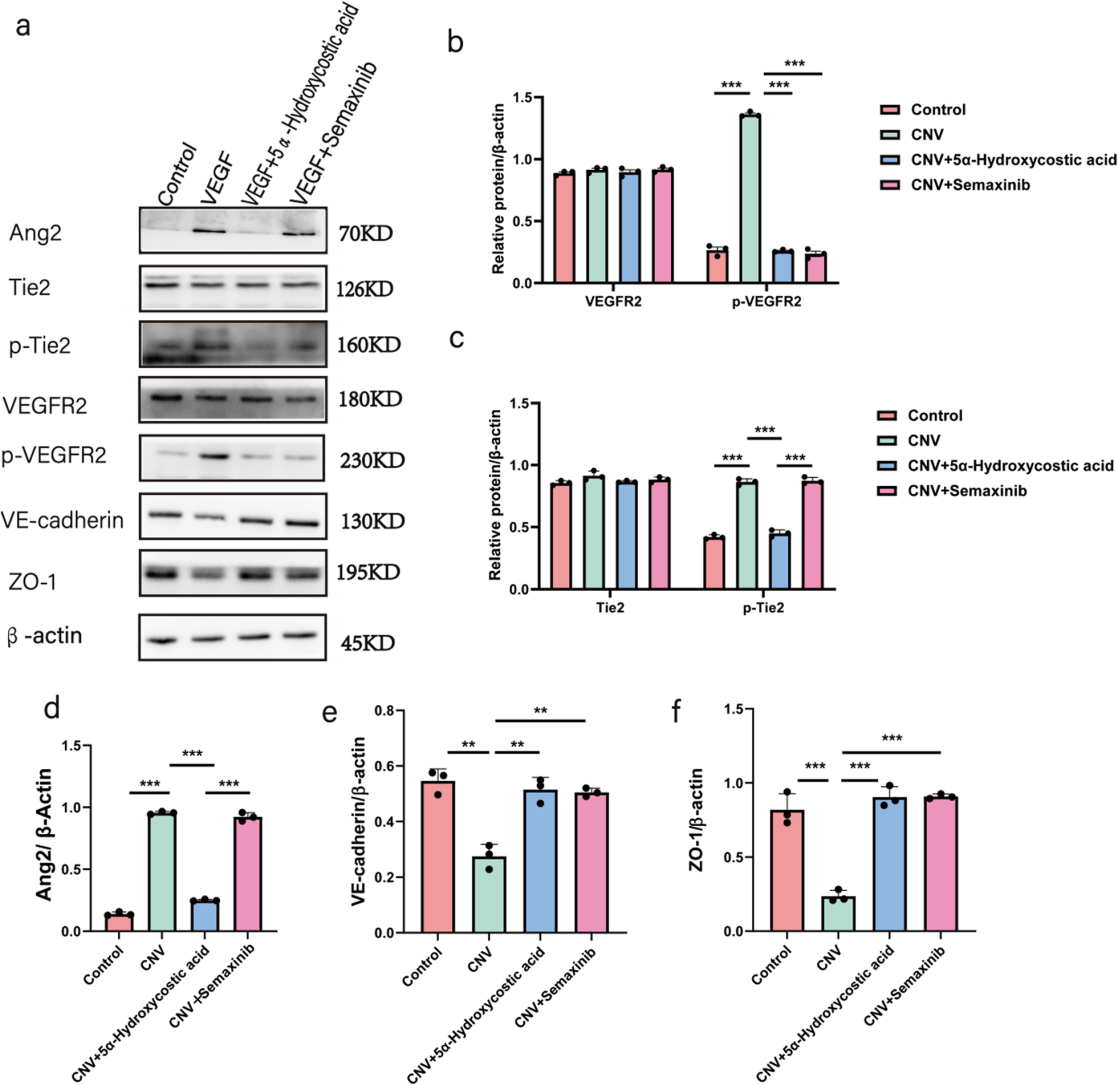
5α-Hydroxycostic acid plays an anti-neovascularization role by regulating VEGF/VEGFR and Ang2/Tie2 signaling pathways. The rats (n = 15) were randomly divided into four groups: control group, CNV group, 5α-Hydroxycostic acid treatment group (CNV + 5α-Hydroxycostic acid), and Semaxinib treatment group. **a**–**f** The protein levels of VEGFR2, p-VEGFR2, Ang2, Tie2, p-Tie2, VE-cadherin and ZO-1 in choroid tissues were determined by western blot. The results are presented as the mean ± SD. n = 3; **P < 0.01, ***P < 0.001

In our in vitro experiments, we demonstrated that 5α-Hydroxycostic acid can reverse the proliferation, migration, and tubule formation of VEGF164-treated endothelial cells, and noted that its inhibition occurs through the VEGF/VEGFR and Ang2/Tie2 pathways. To provide a comprehensive understanding of the therapeutic effect of 5α-Hydroxycostic acid on choroidal neovascularization, we observed the improvement in fundus lesions of rats by establishing a choroidal neovascularization model and administering drug treatment. Figure 5a shows the fluorescein fundus angiography (FFA) results after creating the CNV model. The HE staining results revealed that the structures of the retina, Bruch membrane, and retina pigment epithelium (RPE) layer were disarrayed in the CNV + 0.2% DMSO group. Discontinuous tissue structures were observed in the inner and outer nuclear layers, and abnormally proliferating cells were observed to break the basement membrane of the choroidal vascular network. In the 5α-Hydroxycostic acid-treated group and Semaxinib-treated group, the area and degree of damage to the retina and choroid were significantly reduced (Fig. 5d). To quantify the area of new vessel leakage in each group further, we conducted choroidal patch imaging 14 days after intravitreal injection. The results showed that the choroidal neovascularization area in the 5α-Hydroxycostic acid treatment group and Semaxinib-treated group was significantly smaller than that in the CNV + 0.2% DMSO group (Fig. 5b, c). Moreover, we confirmed that the protein expression in choroidal tissue of rats was consistent with the results of the cell experiments (Fig. 6). In conclusion, these results demonstrate that 5α-Hydroxycostic acid has a positive effect in treating choroidal neovascularization in rats, similar to anti-VEGF therapy. It can effectively reduce vascular leakage and tissue damage, and is expected to be utilized as a new natural drug for treating choroidal neovascularization.

## Discussion

Choroidal neovascularization (CNV) is one of the most severe symptoms of the late stage of wet senile macular disease (wAMD). If left untreated, CNV aggravates, leading to irreversible damage to vision. The occurrence and development of CNV are affected by multiple factors, but the pathogenesis is (Zhou et al. 2020) still unclear. Recently, it has been found that Ang2 and VEGFA cooperate to promote retinal vascular leakage, angiogenesis, and (Khan et al. 2020; Joussen et al. 2021) inflammation. Some studies have demonstrated that VEGF alone blocks the tight junctions between endothelial cells twice as effectively as Ang2, while the combined effect of two growth factors increases permeability three times more than that of VEGF (Peters et al. 2007) alone. Liang et al. discovered that 5α-Hydroxycostic acid, a natural drug, can inhibit the migration of breast cancer cells by interfering with VEGF- and Ang2-related (Liang et al. 2017) pathways. Currently, no research has been conducted on this drug in ophthalmology. We suggested that 5α-Hydroxycostic acid might have an anti-neovascularization effect in the treatment of ophthalmic choroidal neovascularization. In our study, we discovered that 5α-Hydroxycostic acid can effectively inhibit the mRNA expression of Ang2, VEGFR, and Tie2. Meanwhile, Western blotting results proved that 5α-Hydroxycostic acid can inhibit the protein phosphorylation modification process of VEGFR and Tie2, thereby suppressing a series of biological effects that promote angiogenesis. This conclusion was also validated in vivo. In conclusion, studying the role of 5α-Hydroxycostic acid in ocular choroidal neovascularization and its underlying mechanisms is of great significance.

The generation of blood vessels is largely accomplished by the proliferation and migration of endothelial cells, which are regulated by a variety of cytokines, among which VEGF plays a (Woolard et al. 2009) critical role. Increasing evidence suggests that Ang2 expression is closely related to tumour invasion and metastasis in various human cancers. Ang2 promotes tumour angiogenesis through Tie2 signalling and acts synergistically with vascular endothelial growth factor. In colon cancer cell and tumour endothelial cell studies, tumours derived from Ang2-transfected cells not only had more blood vessels but also exhibited higher tumour cell (Ahmad et al. 2001) proliferation. Some researchers have found that increased Ang2 levels are correlated with the severity of neovascular (Ng et al. 2017; Gao and Xu 2008) diseases. These previous studies have shown that Ang2 promotes blood vessel generation, cell proliferation, and cell migration and has a positive effect on the development of the disease. Hence, targeting the Ang2 pathway has become an important approach for treating tumours and angiogenesis. In this study, rat choroidal vascular endothelial cells were cultured in vitro, and we successfully induced the process of CNV formation by adding VEGF to the medium to mimic the abnormal VEGF increase that occurs during CNV formation. Moreover, we applied an appropriate concentration of 5α-Hydroxycostic acid for intervention, and found that it can effectively inhibit the proliferation, migration, and lumen formation of VEGF-treated RCSECs. 5α-Hydroxycostic acid exerts a significant inhibitory effect on CNV in vitro and has a great potential to treat CNV. Adherens junctions (AJs) play a crucial role in forming connections between vascular endothelial (Franke 2009) cells. VE-cadherin and ZO-1 also play important roles in the formation of tight junction (TJ) (Garcia et al. 2018) complexes. Classical cadherin is the primary transmembrane protein found in adhesion junctions (Hartsock and Nelson 2008). Adhesion junctions are responsible for regulating cellular permeability and maintaining local microenvironment stability. Several studies have found that VEGFA can activate FAK, which then activates Src. This degradation of the complex formed by VE-cadherin and tight-junction proteins results in an increase in the size of the endothelial space and ultimately leads to increased vascular permeability (Zhang et al. 2015; Chen et al. 2012). Moreover, VEGFA promotes the expression of ang2 in endothelial cells, which exerts a host of biological effects, including neovascularization and vascular leakage (Fagiani and Christofori 2013; Apte et al. 2019). Morphological animal studies show that injecting 5α-Hydroxycostic acid into the vitreous cavity can inhibit the development of CNV, reduce the area of vascular leakage, and increase the protein expression of VE-cadherin and ZO-1. As a result, we conclude that 5α-Hydroxycostic acid inhibits angiogenesis by enhancing the tight junctions between cells and reducing leakage. Currently, several anti-VEGF drugs in clinical ophthalmology can inhibit the growth of choroidal neovascularization and vascular leakage by preventing VEGF from binding to its receptors. However, not all patients respond equally well to these therapies, leaving them with few other treatment options. This study proves that 5α-Hydroxycostic acid not only inhibits the binding of VEGF to its receptor but also inhibits the activation of the Tie2 pathway by Ang2, thereby reversing the low expression of VE-cadherin and ZO-1.

## Conclusion

These findings suggest that 5α-Hydroxycostic acid has the potential to become a new type of natural anti-neovascularization drug, thus providing a novel approach for the clinical treatment of choroidal neovascularization.

## Data Availability

All data generated or analyzed during this study are included in this article. The datasets used and/or analyzed during the current study are available from the corresponding author on reasonable request.
